# Urinary Levels of 4-Nonylphenol and 4-t-Octylphenol in a Representative Sample of the Korean Adult Population

**DOI:** 10.3390/ijerph14080932

**Published:** 2017-08-18

**Authors:** Hyejin Park, Kisok Kim

**Affiliations:** 1 Department of International Medical Management, Daegu Catholic University, Gyeongsan 38430, Korea; hjpark@cu.ac.kr; 2 College of Pharmacy, Keimyung University, Daegu 42601, Korea

**Keywords:** alkylphenol, biomonitoring, human urine, demographic characteristics

## Abstract

4-Nonylphenol (4-NP) and 4-t-octylphenol (4-t-OP) are xenoestrogen compounds to which humans are exposed via contaminated food, water, and air. This study assessed the body burden of 4-NP and 4-t-OP in Koreans aged 18–69 years using data from the Korean National Human Biomonitoring Survey conducted in 2009. Based on data from 1865 representative Koreans, 83.2% and 91.8% had urinary 4-NP and 4-t-OP concentrations >0.05 ng/mL (limit of detection). Of the Korean adult population, the geometric mean urinary concentrations of 4-NP and 4-t-OP were 3.70 ng/mL (95% confidence interval (CI) = 3.20–4.27) and 0.60 ng/mL (95% CI = 0.55–0.66), respectively. Urine 4-NP concentrations were significantly associated with place of residence and smoking status, whereas urine 4-t-OP concentrations were not correlated with any of the demographic factors. These findings suggest that most Koreans have detectable levels of 4-NP and 4-t-OP in their urine and that the body burden of 4-NP, but not 4-t-OP, varies according to some demographic factors.

## 1. Introduction

Humans are potentially exposed to a wide range of toxic chemicals present in commonly used products and in environmental media. Human biomonitoring surveys of these chemicals are important for determining the mean exposure level of a population, describing geographical differences, identifying high-risk groups, and assessing health risks in a population [1]. Therefore, several countries, including the United States (U.S.) and Germany, have conducted nationally representative biomonitoring surveys that have included analyses of phenolic compounds. Among the phenolic compounds to which humans are most commonly exposed are 4-nonylphenol (4-NP) and 4-t-octylphenol (4-t-OP), human-made alkyl phenols [2,3].

As 4-NP and 4-t-OP are intermediates in the production of alkylphenol ethoxylates (APEs), the manufacture and biodegradation of APEs have been demonstrated to be an important source of 4-NP and 4-t-OP environmental contamination [4]. Because 4-NP and 4-t-OP are widespread environmental contaminants found in wastewater, potable water, rivers, and biota [5,6,7], the general public can be exposed to these chemicals through drinking water, contaminated foods, air inhalation, and dermal absorption [8,9]. As estrogen-mimetic compounds, exposure to 4-NP or 4-t-OP can affect the endocrine system by interacting with estrogen receptors and disrupting normal signaling pathways [10]. Several studies have reported that exposure to these chemicals results in reproductive and developmental toxicity in humans. Exposure to 4-NP induces male infertility by exerting a negative impact on spermatogenesis and sperm quality [11], while maternal urinary concentrations of 4-t-OP are reportedly significantly associated with neonatal size at birth [12].

South Korea conducted a human biomonitoring survey for hazardous materials, which included urinary concentrations of 4-NP and 4-t-OP, among a representative sample of Korean adults aged 18–69 years. Previous biomonitoring studies of phenolic compounds have shown that urinary chemical levels vary significantly according to the population studied, reflecting differences in exposure levels depending on geographical location [13,14]. Furthermore, epidemiological studies have revealed many contributing factors to the body burdens of phenolic compounds, including age, household income, and cigarette smoking [14,15]. Therefore, in this study, we used Korean national survey data to examine urinary 4-NP and 4-t-OP concentrations in Korean adults and to elucidate the demographic characteristics that potentially influence these concentrations.

## 2. Methods

### 2.1. Study Population

The participants for this study were selected from the Korean National Human Biomonitoring Survey (KNHBS). The KNHBS was a population-based, cross-sectional survey representing the adult population (18–69 years of age) residing in the Republic of Korea. We excluded participants with very low or very high urinary creatinine concentrations (<30 mg/dL or >300 mg/dL), because these are defined by the WHO as too dilute or too concentrated for adequate analysis [16]. A total of 1865 subjects completed interviews without missing data, provided urine samples, and were included in the analyses. This study was supervised by the Korean Food and Drug Administration, and the study protocol was approved by the Asan Medical Center Institutional Review Board (IRB approval # 2009-0369). The study was conducted in accordance with the ethical principles for medical research involving human subjects as defined by the Declaration of Helsinki. Study participants provided written, informed consent.

### 2.2. Data Collection

Data about participants’ sex, age, education, income, cigarette smoking, and current residence were collected during face-to-face interviews. Education was categorized as less than a high school diploma, high school diploma, and college or higher. Income was classified into four groups based on monthly household income. Cigarette smoking status was defined as never, former, or current. Body mass index (BMI) was calculated as weight (kg) divided by height squared (m^2^). Then, the participants were divided into four groups: underweight (BMI < 18.5 kg/m^2^), normal weight (BMI = 18.5–22.9 kg/m^2^), overweight (BMI = 23.0–25.0 kg/m^2^), or obese (BMI ≥25.0 kg/m^2^) according to the World Health Organization’s classification system for Asian populations [17]. Spot urine samples were collected at different times throughout the day, and creatinine adjustments were used to correct for urine dilution. The urine samples were analyzed at the Korean Institute of Science and Technology (Seoul, Korea) using liquid-liquid extraction and gas chromatography-mass spectrometry [18]. In brief, the urine samples were thawed at room temperature and vortex-mixed. After gentle mixing, the samples were added to 50 μL of glucuronidase/arylsulfatase solution (in 0.2 M sodium acetate buffer, pH 5.2), and hydrolysis was allowed to proceed at 55 °C for 3 h. After cooling to room temperature, the samples were added to a 5% K_2_CO_3_ solution and extracted with methyl tert-butyl ether. The dried extracts were derivatized with 50 μL of a bis-(trimethylsilyl) trifluoroacetamide/trimethylchlorosilane (100:1, v/v) mixture at 60 °C for 30 min. The samples were analyzed with mass-selective detectors (models 6890 Gas Chromatograph and 5975; Agilent Technologies, Palo Alto, CA, USA) connected to an Ultra-2 column (25 m × 0.2 mm internal diameter, 0.33-μm film thickness; Agilent Technologies). As reference materials, 4-NP and 4-t-OP were obtained from Sigma-Aldrich (Steinheim, Germany), with a purity of >99%. Recovery was assessed by adding known amounts of the standards, and was in the range of 83.5–111.1% for 4-NP and 89.5–102.8% for 4-t-OP. The intra- and interday accuracy and precision were examined by analyzing the four analytes in seven replicates during a single day and on five consecutive days. The intraday accuracy for 4-NP was 95.0–109.5% with a precision of 5.3–14.6%, whereas its interday accuracy was 98.7–103.4% with a precision of 3.0–6.3%. The intraday accuracy of 4-t-OP was 88.0–103.0% with a precision of 6.0–10.1%, whereas its interday accuracy was 96.3–101.1% with a precision of 3.3–6.1%. Linearity in these analytes was checked from 0.1 (limit of quantification (LOQ)) to 200 ng/mL, with correlation coefficients of 0.9971 and 1.0000 for 4-NP and 4-t-OP, respectively. The limit of detection (LOD) and the LOQ for each analyte under the chromatographic conditions were determined at signal-to-noise ratios of 3 and 10, respectively. The LOD and LOQ were 0.05 ng/mL and 0.20 ng/mL, respectively, for 4-NP and 0.05 ng/mL and 0.10 ng/mL, respectively, for 4-t-OP. Individuals whose urinary concentration fell below the LOD were assigned a value of LOD/2 [19]. Creatinine levels were measured by means of a kinetic Jaffé method using a Hitachi 7600 auto-analyzer (Hitachi, Tokyo, Japan).

### 2.3. Statistical Analyses

We used selected percentile and maximum values to describe the distributions of 4-NP and 4-t-OP levels. We also calculated geometric means with 95% confidence intervals (CIs) for urinary 4-NP and 4-t-OP concentrations by taking the antilog of the mean of the natural log-transformed values. Sample weights were applied to adjust for the differential selection probabilities of selected participants to calculate weighted geometric means. We fitted multiple linear regressions of the log-transformed concentrations with the weights for the predictor variables. The exponentiated model coefficients represent proportional changes in the arithmetic mean associated with each level of the predictor relative to a referent level, adjusting for the other predictors in the model. The difference between the two demographic subgroups in mean values and the presence of a linear trend among subgroups were evaluated by a survey t-test and by defining a linear contrast in each of the general linear models, respectively. All statistical analyses were conducted using SAS 9.4 computer software (SAS Institute, Cary, NC, USA).

## 3. Results

A total of 1865 eligible subjects participated in the study, yielding a participation rate of 87.1%. The mean age of the subjects was 45.5 years, and 57.0% of the participants were female (Table 1).

Selected percentiles of 4-NP levels in the participants’ urine samples are presented in Table 2; 83.2% of sample values were above the LOD and ranged between 0.05 ng/mL (LOD) and 4477.0 ng/mL. Urinary 4-NP concentration was not clearly associated with sex. However, the 4-NP concentrations of participants aged 40–49 years were lower than those of other age groups at most percentile points.

Table 3 shows the selected percentiles of 4-t-OP levels in the participants’ urine samples by sex and age. 4-t-OP was detected above the LOD (0.05 ng/mL) in 1713 of the 1865 (91.8%) participants, with total concentrations ranging from 0.05 ng/mL (LOD) to 988.7 ng/mL. Similar to 4-NP, the 4-t-OP concentration was not obviously associated with sex. Although there were apparent decreases in the 4-t-OP levels of participants aged 50–59 years compared with other age groups at most of the percentiles, the creatinine-adjusted 4-t-OP concentrations of this age group were not significantly different from those of participants in other age groups at most percentile points.

The population-weighted geometric mean urinary 4-NP concentration in Korean adults aged 18–69 years was 3.70 ng/mL (95% CI = 3.20–4.27; Table 4). Among demographic characteristics, geometric mean urinary 4-NP concentrations were significantly correlated with place of residence; subjects living in urban areas had higher urinary 4-NP concentrations than those living in rural areas (*p* = 0.005). After adjusting for potential covariates, the adjusted proportional changes in mean 4-NP levels still changed significantly with place of residence (*p* = 0.020). In addition, compared with never smokers, current smokers showed an adjusted proportional change of 1.73 (95% CI = 1.08–2.76). However, geometric means and adjusted proportional changes in 4-NP levels were not significantly correlated with other demographic variables such as sex, age, BMI, educational level, or income.

The population-weighted geometric mean urinary concentration for 4-t-OP was 0.60 ng/mL (95% CI = 0.55–0.66) (Table 5). The analysis of geometric means and adjusted proportional changes in 4-t-OP levels according to demographic factors showed that sex, age, BMI, education level, income, cigarette smoking, and place of residence were not significantly associated with urinary 4-t-OP levels.

## 4. Discussion

Our results revealed that the geometric mean of the urinary 4-NP level for Korean adults was 3.70 ng/mL. The US National Health and Nutrition Examination Survey (NHANES) III reported that the geometric mean of urinary 4-NP level was below the LOD (0.1 ng/mL) for the US adult population [20]. On the other hand, several studies conducted in Taiwan showed that the geometric mean of urinary 4-NP concentration was 2.90–4.10 ng/mL for Taiwanese pregnant women with a mean age range of 31.0–33.4 years [21,22,23,24]. Given that 4-NP levels do not significantly differ by sex, the geometric mean of urinary 4-NP concentration in the Korean population is assumed to be similar to that in the Taiwanese population and much higher than that in the U.S. population.

The geometric mean of the urinary 4-t-OP level for Korean adults was 0.60 ng/mL, which is higher than that in the U.S. population. The 2003–2004 NHANES reported a urinary geometric mean 4-t-OP concentration of 0.3 ng/mL for the U.S. population aged 6 years and over [13]. A possible factor influencing the difference is that our study had a lower LOD (0.05 ng/mL) and higher percentage greater than the LOD (91.8%) than did the NHANES study (LOD = 0.2 ng/mL, percentage greater than the LOD, 57.4%), which would yield more precise percentiles and geometric means compared with studies with a higher LOD. Other studies have reported that the geometric mean 4-t-OP concentrations in the urine of Chinese men and women were 0.60 ng/mL and 0.90 ng/mL, respectively [12,25], which is similar to those of the Korean general population shown in this study.

4-NP and 4-t-OP are still used and are commonly detected in environmental media in Asian countries, including Korea, Taiwan, and China, whereas the U.S. has initiated a phase-out of these chemicals [26,27,28]. Therefore, the difference in body burden of 4-NP and 4-t-OP between U.S. and Asian populations may be attributed to a difference in exposure levels. Because a single spot-urine sample per participant was analyzed, within-person variability in urinary concentrations over time may be a limitation of this study. However, estimation of mean population levels based on one spot sample per participant is considered to be a useful approach in cross-sectional studies [13].

Lifestyle factors, including smoking and place of residence, are related to urinary concentrations of some phenolic compounds [29,30,31,32,33], although the biological basis of these associations needs to be elucidated. In this study, subjects living in urban regions had higher urinary 4-NP concentrations than those living in rural regions, and adjusted proportional changes showed that cigarette smoking was significantly associated with increased urinary 4-NP levels. These results indicate that place of residence and smoking emerged as important factors influencing urinary concentrations of 4-NP, which suggests the possibility of variations in exposure to 4-NP based on place of residence and smoking status.

The strength of this study is that it is the first study to assess the body burdens of 4-NP and 4-t-OP and their association with demographic characteristics among Korean adults using nationally representative data. Another strength of this study is that we used sample weights to obtain the urinary 4-NP and 4-t-OP levels in the Korean population, which may have led to more precise estimates of nationally representative values for adult Koreans. However, these findings highlight the need for additional research to identify pathways of human exposure and to evaluate the potential effects of exposure to these chemicals on health.

## 5. Conclusions

Using nationally representative data, we found that a considerable portion of the Korean general population aged 18–69 years has urinary levels of 4-NP and 4-t-OP above 0.05 ng/mL. The geometric mean urinary levels of 4-NP and 4-t-OP were 3.70 ng/mL (95% CI = 3.20–4.27) and 0.60 ng/mL (95% CI = 0.55–0.66), respectively. Among the sociodemographic characteristics studied, place of residence and cigarette smoking were significant factors of the urinary 4-NP concentration. These findings suggest the need for policies to evaluate the potential health effects on high-risk groups and to reduce human exposure to 4-NP and 4-t-OP.

## Figures and Tables

**Table 1:** General characteristics of study participants.

Characteristics	*N* (%)
Total	1865 (100.0)
Women	1063 (57.0)
Age (years)	
18–29	247 (13.2)
30–39	412 (22.1)
40–49	454 (24.3)
50–59	430 (23.1)
60–69	322 (17.3)
Body mass index (BMI), kg/m^2^	
<18.5	56 (3.0)
18.5–22.9	816 (43.7)
23.0–24.9	453 (24.3)
≥25.0	540 (29.0)
Education	
<High school	529 (28.4)
High school	705 (37.8)
>High school	631 (33.8)
Income, U.S. $/month	
<910	399 (21.4)
910–2729	894 (47.9)
2730–4550	423 (22.7)
>4550	149 (8.0)
Cigarette smoking status	
Never	1232 (66.1)
Former	228 (12.2)
Current	405 (21.7)
Place of residence	
Rural	448 (24.0)
Urban	1417 (76.0)

**Table 2:** Selected urine concentration percentiles of 4-nonylphenol in the Korean population aged 18–69 years, by sex and age subgroups.

Variable	*N*	% > LOD *	Percentile					Max
			25th	50th	75th	90th	95th	
All	1865	83.2	1.02 (0.96)	8.10 (7.50	21.9 (23.0)	61.7 (64.1)	137.4 (145.6)	4477.0 (10,435.8)
Sex								
Male	802	82.9	0.95 (0.72)	7.94 (6.27)	20.9 (17.0)	66.3 (56.3)	150.9 (132.1)	781.8 (1541.9)
Female	1063	83.4	1.02 (1.08)	8.19 (8.62)	22.9 (28.2)	58.3 (72.2)	128.0 (156.5)	4477.0 (10,435.8)
Age (years)								
18–29	247	82.6	0.64 (0.41)	9.83 (6.62)	22.7 (20.6)	78.8 (61.4)	182.4 (131.6)	658.0 (974.8)
30–39	412	83.0	1.79 (1.46)	8.99 (8.99)	24.9 (24.9)	62.5 (82.4)	186.0 (176.2)	1039.1 (1135.7)
40–49	454	82.6	0.35 (0.44)	6.42 (6.46)	17.2 (18.9)	49.1 (52.2)	110.0 (111.3)	781.8 (1541.9)
50–59	430	83.5	1.02 (1.08)	8.19 (7.76)	22.9 (25.8)	60.2 (63.1)	117.0 (152.6)	4477.0 (10,435.8)
60–69	322	84.5	1.10 (1.28)	8.50 (8.47)	23.0 (29.3)	66.0 (71.1)	140.0 (149.8)	654.6 (944.1)

* Limit of detection (LOD) = 0.05 ng/mL; Percentile values are expressed as volume-based concentrations (ng/mL), and creatinine-adjusted concentrations (μg/g creatinine) are in parentheses.

**Table 3:** Selected urine concentration percentiles of 4-t-octylphenol in the Korean population aged 18–69 years, by sex and age subgroups.

Variable	*N*	% > LOD *	Percentile					Max
			25th	50th	75th	90th	95th	
All	1865	91.8	0.17 (0.17)	0.77 (0.71)	1.80 (1.94)	4.40 (4.86)	8.00 (8.64)	988.7 (645.4)
Sex								
Male	802	92.6	0.16 (0.15)	0.74 (0.56)	1.80 (1.39)	4.10 (3.60)	8.20 (6.89)	356.3 (240.3)
Female	1063	91.3	0.18 (0.20)	0.81 (0.87)	1.90 (2.37)	4.70 (5.69)	7.80 (10.22)	988.7 (645.4)
Age (years)								
18–29	247	90.7	0.14 (0.12)	0.83 (0.60)	1.70 (1.29)	3.80 (3.50)	7.40 (5.73)	47.1 (24.3)
30–39	412	91.3	0.19 (0.18)	0.81 (0.69)	1.96 (2.04)	4.19 (5.31)	7.61 (8.38)	988.7 (645.4)
40–49	454	89.9	0.15 (0.15)	0.86 (0.84)	1.91 (2.07)	5.13 (5.69)	9.22 (8.54)	356.3 (144.6)
50–59	430	93.3	0.13 (0.15)	0.66 (0.65)	1.60 (1.84)	3.80 (4.05)	6.50 (6.97)	73.1 (104.8)
60–69	322	94.4	0.20 (0.22)	0.77 (0.76)	2.04 (2.06)	5.44 (6.30)	9.66 (13.37)	336.8 (232.7)

* LOD = 0.05 ng/mL; Percentile values are expressed as volume-based concentrations (ng/mL), and creatinine-adjusted concentrations (μg/g creatinine) are in parentheses.

**Table 4:** Population-weighted geometric means and adjusted proportional changes in urinary 4-nonylphenol concentrations by demographic characteristics in the Korean population aged 18–69 years.

Variable	*N*	Geometric Mean (95% CI), ng/mL	*p*-Value ^a^	Adjusted Proportional Change (95% CI) ^b^	*p*-Value
Total		3.70 (3.20–4.27)		-	
Sex					
Male	802	3.67 (2.97–4.54)	0.9253	0.65 (0.42–1.00)	0.052
Female	1063	3.72 (3.06–4.53)		1.00 (reference)	
Age (years)					
18–29	247	3.50 (2.40–5.10)	0.787	1.00 (reference)	0.073
30–39	412	4.36 (3.27–5.81)		1.32 (0.82–2.11)	
40–49	454	3.08 (2.35–4.04)		1.04 (0.64–1.70)	
50–59	430	3.91 (2.96–5.15)		1.44 (0.83–2.49)	
60–69	322	3.98 (2.91–5.43)		1.83 (1.00–3.33)	
BMI					
<18.5	56	3.78 (1.53–9.37)	0.838	1.00 (reference)	0.974
18.5–22.9	816	3.65 (2.93–4.56)		1.00 (0.41–2.44)	
23.0–24.9	453	4.53 (3.39–6.05)		1.29 (0.52–3.19)	
≥25.0	540	3.20 (2.47–4.13)		0.91 (0.37–2.21)	
Education					
<High school	529	3.48 (2.69–4.51)	0.268	1.00 (reference)	0.349
High school	705	3.21 (2.53–4.07)		0.91 (0.61–1.37)	
>High school	631	4.24 (3.35–5.36)		1.27 (0.77–2.11)	
Income (U.S. $/month)					
<910	399	2.64 (1.87–3.72)	0.068	1.00 (reference)	0.122
910–2729	894	3.99 (3.26–4.89)		1.53 (0.98–2.39)	
2730–4550	423	3.65 (2.72–4.90)		1.40 (0.83–2.36)	
>4550	149	4.84 (2.92–8.01)		1.77 (0.91–3.41)	
Cigarette smoking status					
Never	1232	3.34 (2.79–4.00)	0.136	1.00 (reference)	0.023
Former	228	4.25 (2.99–6.03)		1.61 (0.99–2.63)	
Current	405	4.39 (3.21–5.99)		1.73 (1.08–2.76)	
Place of residence					
Rural	448	2.49 (1.85–3.35)	0.005	0.66 (0.47–0.93)	0.020
Urban	1417	4.03 (3.42–4.75)		1.00 (reference)	

^a^ *p* determined by survey *t*-test or linear trend test; ^b^ The exponentiated *β*-coefficient from a log-linear multiple regression that included all covariates in the table and urinary creatinine concentration.

**Table 5:** Population-weighted geometric means and adjusted proportional changes in urinary 4-t-octylphenol concentrations by demographic characteristics in the Korean population aged 18–69 years.

Variable	*N*	Geometric Mean (95% CI), ng/mL	*p*-Value ^a^	Adjusted Proportional Change (95% CI) ^b^	*p*-Value
Total		0.60 (0.55–0.66)		-	
Sex					
Male	802	0.57 (0.50–0.65)	0.193	0.77 (0.59–1.01)	0.057
Female	1063	0.64 (0.57–0.72)		1.00 (reference)	
Age (years)					
18–29	247	0.57 (0.45–0.72)	0.551	1.00 (reference)	0.189
30–39	412	0.63 (0.53–0.75)		1.22 (0.91–1.64)	
40–49	454	0.63 (0.52–0.75)		1.27 (0.92–1.77)	
50–59	430	0.52 (0.45–0.61)		1.09 (0.77–1.54)	
60–69	322	0.69 (0.57–0.84)		1.44 (0.98–2.11)	
BMI					
<18.5	56	0.97 (0.57–1.63)	0.087	1.00 (reference)	0.107
18.5–22.9	816	0.58 (0.51–0.66)		0.63 (0.38–1.03)	
23.0–24.9	453	0.63 (0.52–0.76)		0.67 (0.40–1.14)	
≥25.0	540	0.59 (0.50–0.70)		0.63 (0.37–1.06)	
Education					
<High school	529	0.61 (0.52–0.71)	0.759	1.00 (reference)	0.234
High school	705	0.57 (0.50–0.67)		1.04 (0.80–1.35)	
>High school	631	0.63 (0.54–0.73)		1.22 (0.88–1.70)	
Income (U.S. $/month)					
<910	399	0.63 (0.51–0.80)	0.426	1.00 (reference)	0.525
910–2729	894	0.60 (0.53–0.68)		0.96 (0.72–1.29)	
2730–4550	423	0.54 (0.45–0.64)		0.85 (0.60–1.21)	
>4550	149	0.78 (0.55–1.12)		1.21 (0.77–1.91)	
Cigarette smoking status					
Never	1232	0.61 (0.55–0.69)	0.569	1.00 (reference)	0.638
Former	228	0.62 (0.50–0.78)		1.17 (0.86–1.60)	
Current	405	0.57 (0.48–0.69)		1.07 (0.80–1.44)	
Place of residence					
Rural	448	0.70 (0.59–0.83)	0.076	1.21 (0.98–1.48)	0.075
Urban	1417	0.58 (0.53–0.65)		1.00 (reference)	

^a^ *p* determined by survey *t*-test or linear trend test; ^b^ The exponentiated *β*-coefficient from a log-linear multiple regression that included all covariates in the table and urinary creatinine concentration.

## References

[1] Draper W.M. (2001). Biological monitoring: Exquisite research probes, risk assessment, and routine exposure measurement. Anal. Chem..

[2] Van Miller J.P., Staples C.A. (2005). Review of the potential environmental and human health-related hazards and risks from long-term exposure to p-tert-octylphenol. Hum. Ecol. Risk Assess..

[3] Vazquez-Duhalt R., Marquez-Rocha F., Ponce E., Licea A.F., Viana M.T. (2005). Nonyphenol, an integrated vision of a pollutant. Appl. Ecol. Environ. Res..

[4] David A., Fenet H., Gomez E. (2009). Alkylphenols in marine environments: Distribution monitoring strategies and detection considerations. Mar. Pollut. Bull..

[5] Hawker D.W., Cumming J.L., Neale P.A., Bartkow M.E., Escher B.I. (2011). A screening level fate model of organic contaminants from advanced water treatment in a potable water supply reservoir. Water Res..

[6] Gatidou G., Vassalou E., Thomaidis N.S. (2010). Bioconcentration of selected endocrine disrupting compounds in the *Mediterranean mussel*, *Mytilus galloprovincialis*. Mar. Pollut. Bull..

[7] Stasinakis A.S., Gatidou G., Mamais D., Thomaidis N.S., Lekkas T.D. (2008). Occurrence and fate of endocrine disrupters in Greek sewage treatment plants. Water Res..

[8] Ahel M., McEvoy J., Giger W. (1993). Bioaccumulation of the lipophilic metabolites of nonionic surfactants in freshwater organisms. Environ. Pollut..

[9] Clark L.B., Rosen R.T., Hartman T.G., Louis J.B., Suffet I., Lippincott R., Rosen J.D. (1992). Determination of alkylphenol ethoxylates and their acetic acid derivatives in drinking water by particle beam liquid chromatography/mass spectrometry. Int. J. Environ. Anal. Chem..

[10] Laws S.C., Carey S.A., Ferrell J.M., Bodman G.J., Cooper R.L. (2000). Estrogenicactivity of octylphenol, nonylphenol, bisphenol A and methoxychlor in rats. Toxicol. Sci..

[11] Noorimotlagh Z., Haghighi N.J., Ahmadimoghadam M., Rahim F. (2017). An updated systematic review on the possible effect of nonylphenol on male fertility. Environ. Sci. Pollut. Res. Int..

[12] Lv S., Wu C., Lu D., Qi X., Xu H., Guo J., Liang W., Chang X., Wang G., Zhou Z. (2016). Birth outcome measures and prenatal exposure to 4-tert-octylphenol. Environ. Pollut..

[13] Calafat A.M., Ye X., Wong L.Y., Reidy J.A., Needham L.L. (2008). Exposure of the U.S. population to bisphenol A and 4-tertiary-octylphenol: 2003–2004. Environ. Health Perspect..

[14] Kim K., Park H., Yang W., Lee J.H. (2011). Urinary concentrations of bisphenol A and triclosan and associations with demographic factors in the Korean population. Environ. Res..

[15] Calafat A.M., Ye X., Wong L.Y., Reidy J.A., Needham L.L. (2008). Urinary concentrations of triclosan in the U.S. population: 2003–2004. Environ. Health Perspect..

[16] Barr D.B., Wilder L.C., Caudill S.P., Gonzalez A.J., Needham L.L., Pirkle J.L. (2005). Urinary creatinine concentrations in the U.S. population: implications for urinary biologic monitoring measurements. Environ. Health Perspect..

[17] World Health Organization The Asian-Pacific Perspective: Redefining Obesity and Its Treatment. http://www.wpro.who.int/nutrition/documents/docs/Redefiningobesity.pdf.

[18] Kim K., Park H., Lee J.H. (2014). Urinary concentrations of trichlorophenols in the Korean adult population: Results of the National Human Biomonitoring Survey 2009. Environ. Sci. Pollut. Res. Int..

[19] Cole S.R., Chu H., Nie L., Schisterman E.F. (2009). Estimating the odds ratio when exposure has a limit of detection. Int. J. Epidemiol..

[20] Calafat A.M., Kuklenyik Z., Reidy J.A., Caudill S.P., Ekong J., Needham L.L. (2005). Urinary concentrations of bisphenol A and 4-nonylphenol in a human reference population. Environ. Health Perspect..

[21] Huang Y.F., Pan W.C., Tsai Y.A., Chang C.H., Chen P.J., Shao Y.S., Tsai M.S., Hou J.W., Lu C.A., Chen M.L. (2017). Concurrent exposures to nonylphenol, bisphenol A, phthalates, and organophosphate pesticides on birth outcomes: A cohort study in Taipei, Taiwan. Sci. Total Environ..

[22] Tsai M.S., Chang C.H., Tsai Y.A., Liao K.W., Mao I.F., Wang T.H., Hwang S.M., Chang Y.J., Chen M.L. (2013). Neonatal outcomes of intrauterine nonylphenol exposure—A longitudinal cohort study in Taiwan. Sci. Total Environ..

[23] Wang P.W., Chen M.L., Huang L.W., Yang W., Wu K.Y., Huang Y.F. (2015). Nonylphenol exposure is associated with oxidative and nitrative stress in pregnant women. Free Radic. Res..

[24] Wang P.W., Chen M.L., Huang L.W., Yang W., Wu K.Y., Huang Y.F. (2015). Prenatal nonylphenol exposure, oxidative and nitrative stress, and birth outcomes: A cohort study in Taiwan. Environ. Pollut..

[25] Qin Y., Chen M., Wu W., Xu B., Tang R., Chen X., Du G., Lu C., Meeker J.D., Zhou Z. (2013). Interactions between urinary 4-tert-octylphenol levels and metabolism enzyme gene variants on idiopathic male infertility. PLoS ONE.

[26] Li X., Ying G.G., Zhao J.L., Chen Z.F., Lai H.J., Su H.C. (2013). 4-Nonylphenol, bisphenol-A and triclosan levels in human urine of children and students in China, and the effects of drinking these bottled materials on the levels. Environ. Int..

[27] Asimakopoulos A.G., Thomaidis N.S., Koupparis M.A. (2012). Recent trends in biomonitoring of bisphenol A, 4-t-octylphenol, and 4-nonylphenol. Toxicol. Lett..

[28] Yu C.J., Du J.C., Chiou H.C., Yang S.H., Liao K.W., Yang W., Chung M.Y., Chien L.C., Hwang B., Chen M.L. (2016). Attention deficit/hyperactivity disorder and urinary nonylphenol levels: A case-control study in Taiwanese children. PLoS ONE.

[29] Schettgen T., Alt A., Dewes P., Kraus T. (2015). Simple and sensitive GC/MS-method for the quantification of urinary phenol, o- and m-cresol and ethylphenols as biomarkers of exposure to industrial solvents. J. Chromatogr. B Analyt. Technol. Biomed. Life Sci..

[30] Arbuckle T.E., Marro L., Davis K., Fisher M., Ayotte P., Bélanger P., Dumas P., LeBlanc A., Bérubé R., Gaudreau É. (2015). Exposure to free and conjugated forms of bisphenol A and triclosan among pregnant women in the MIREC cohort. Environ. Health Perspect..

[31] Geens T., Bruckers L., Covaci A., Schoeters G., Fierens T., Sioen I., Vanermen G., Baeyens W., Morrens B., Loots I. (2014). Determinants of bisphenol A and phthalate metabolites in urine of Flemish adolescents. Environ. Res..

[32] Engel L.S., Buckley J.P., Yang G., Liao L.M., Satagopan J., Calafat A.M., Matthews C.E., Cai Q., Ji B.T., Cai H. (2014). Predictors and variability of repeat measurements of urinary phenols and parabens in a cohort of Shanghai women and men. Environ. Health Perspect..

[33] Larsson K., Ljung Björklund K., Palm B., Wennberg M., Kaj L., Lindh C.H., Jönsson B.A., Berglund M. (2014). Exposure determinants of phthalates, parabens, bisphenol A and triclosan in Swedish mothers and their children. Environ. Int..

